# Effect of early antibiotic treatment strategy on prognosis of acute pancreatitis

**DOI:** 10.1186/s12876-023-03070-1

**Published:** 2023-12-09

**Authors:** Yi Wen, Lili Xu, Dayi Zhang, Wenwu Sun, Zaiqian Che, Bing Zhao, Ying Chen, Zhitao Yang, Erzhen Chen, Tongtian Ni, Enqiang Mao

**Affiliations:** Department of Emergency, Ruijin Hospital, Shanghai Jiao Tong University School of Medicine, Shanghai, China

**Keywords:** Acute pancreatitis, Early stage, Antibiotic treatment strategy, Prognosis

## Abstract

**Background:**

Antibiotic use in the early stages of acute pancreatitis is controversial. The purpose of this study was to investigate the effect of early antibiotic application on the prognosis of acute pancreatitis (AP).

**Materials and methods:**

Clinical data of patients with primary AP admitted to our emergency ward within 72 hours of onset were retrospectively collected from January 2016 to December 2020. We classified patients with acute pancreatitis according to etiology and disease severity, and compared the differences in hospital stay, laparotomy rate, and in-hospital mortality among AP patients who received different antibiotic treatment strategies within 72 hours of onset.

**Results:**

A total of 1134 cases were included, with 681 (60.1%) receiving early antibiotic treatment and 453 (39.9%) not receiving it. There were no significant differences in baseline values and outcomes between the two groups. In subgroup analysis, patients with biliary severe acute pancreatitis (SAP) who received early antibiotics had lower rates of laparotomy and invasive mechanical ventilation, as well as shorter hospital stays compared to those who did not receive antibiotics. In logistic regression analysis, the early administration of carbapenem antibiotics in biliary SAP patients was associated with a lower in-hospital mortality rate. Early antibiotic use in biliary moderate-severe acute pancreatitis (MSAP) reduced hospital stays and in-hospital mortality. Quinolone combined with metronidazole treatment in biliary mild acute pancreatitis (MAP) shortened hospital stays. Early antibiotic use does not benefit patients with non-biliary AP.

**Conclusion:**

Strategies for antibiotic use in the early stages of AP need to be stratified according to cause and disease severity.

## Introduction

AP is a condition that involves the activation of pancreatic enzymes [1], resulting in local inflammation of the pancreas. This can lead to self-digestion of pancreatic tissue, as well as edema, bleeding, and necrosis. In severe cases, it may even progress to multiple organ dysfunction or systemic inflammatory response syndrome. There are several causes of acute pancreatitis, with gallstones being the most common (accounting for 45% of cases) followed by alcohol abuse (20%) [2, 3].

Fortunately, acute pancreatitis is often self-limited, with more than two-thirds of patients recovering within a week. However, around one-third of patients may experience complications, both local and systemic. In severe cases, mortality rates can be as high as 10–30%, with infectious complications being a leading cause of death (accounting for 80% of cases) [4, 5]. In particular, patients with severe acute pancreatitis are at risk of developing infection with pancreatic and peripancreatic necrosis, which can lead to further organ dysfunction [6]. As a result, appropriate treatment of infectious necrosis is critical in reducing morbidity and mortality associated with AP.

Infectious complications are a significant concern in the management of AP, as the control of infection can have a significant impact on patient outcomes [7–10]. To ensure the best possible outcome for patients, it is crucial to make an early and accurate diagnosis of infectious pancreatic necrosis. However, this can be challenging in clinical practice, and as such, the judicious use of antibiotics is of utmost importance.

Although the early stages of AP are typically considered sterile, many clinicians still choose to use antibiotics prophylactically. However, this practice is not recommended by the current international consensus due to concerns about the development of resistant flora and limited treatment options in cases of infection [11]. A systematic review demonstrated [12] that prophylactic use of antibiotics reduced the infection rate of acute pancreatitis, primarily in cases of extra-pancreatic infections, without significant impact on infected pancreatic necrosis (IPN) and mortality rates. Although Cochrane analyses [13] have concluded that prophylactic use of antibiotics does not reduce pancreatic necrosis infections, a small number of studies have shown [14, 15] that patients with SAP with necrotic areas greater than 30% may benefit from prophylactic antibiotic use.

There is currently no consensus on the optimal timing and duration of anti-infective treatment for AP, and the threat of multidrug-resistant bacteria further complicates treatment decisions. As such, there is a need for research on the optimal timing and selection of antibiotics in the treatment of AP.

This study aims to retrospectively analyze the impact of antibiotic treatment and selection on the prognosis of AP with different etiologies and disease severity. By doing so, we hope to shed light on the optimal approach to antibiotic treatment in these cases.

## Methods

### Study subjects

This retrospective study collected data on all patients who were admitted to the emergency ward of Ruijin Hospital with initial AP between January 2016 and December 2020. The inclusion criteria for this study were: 1) the ages from 18 to 75; 2) meeting the 2012 Atlanta diagnostic criteria for AP; 3) being admitted within 72 hours of onset; 4) being an incipient patient. 5)Following admission, patients get continuous antibiotic medication for at least 72 hours. The exclusion criteria were: 1) being pregnant or lactating; 2) having a malignant tumor; 3) having pancreatitis caused by endoscopic retrograde cholangiopancreatography (ERCP); 4) having autoimmune diseases; and 5) having chronic organ dysfunction of the heart, lungs, liver, kidneys, or blood system before admission. All patients were managed according to the diagnosis and treatment protocol for AP, which included the following interventions: fasting, gastrointestinal decompression, controlled fluid resuscitation, bowel dredging, early enteral nutrition, and maintenance of homeostasis. This study was approved by the Ethics Committee of Ruijin Hospital Affiliated with Shanghai Jiao Tong University School of Medicine and was granted an exemption from the requirement for informed consent.

### Clinical variables

Through the computer center, electronic medical record data of patients during hospitalization were retrieved, including Age, Sex, Body Mass Index (BMI), Comorbidity, Disease severity grade, Laboratory indicators on admission: leukocyte count, procalcitonin (PCT), C-reactive protein (CRP), Platelet count, Serum amylase, Alanine aminotransferase, Total bilirubin, Creatinine, Calcium, Glucose, Potassium, and Triglycerides. Data were collected on the severity of AP, local and systemic complications, and organ failure according to the revised Atlanta classification. The modified Marshall score, Acute Physiological and Chronic Health Assessment (APACHE II) score, Acute Pancreatitis Severity Index (BISAP) score, and Computed Tomography Severity Index (CTSI) were calculated within 72 hours after admission. Invasive mechanical ventilation rate, acute kidney injury rate, blood purification rate, length of hospital stays, surgical intervention rate, and mortality were compared among different antibiotic strategies treatment within 72 hours of onset in patients with AP.

### Definitions

AP is defined according to the 2012 International Consensus on Acute Pancreatitis in Atlanta [1]. Diagnostic criteria include the following three criteria:

1. Persistent upper abdominal pain.
2. Serum amylase and/or lipase concentrations that are at least three times higher than the normal upper limit.
3. Abdominal imaging findings that are consistent with the imaging changes of AP. AP is diagnosed if two of the three criteria are met.

The etiology of AP is classified as follows:

1. Biliary AP is characterized by elevated total bilirubin and/or aminotransferase (alanine aminotransferase and aspartate aminotransferase) within 72 hours of onset. Imaging confirms the presence of gallstones, common bile duct obstruction, duodenal diverticulum, or common bile duct cyst. Other risk factors are excluded [16].
2. Hyperlipidemic acute pancreatitis (HTGAP) is characterized by a serum triglyceride level exceeding 11.3 mmol/L (1000 mg/dL) upon admission or previous history of hyperlipidemic diseases, and a fasting triglyceride level exceeding 5.65 mmol/L within 72 hours of onset, while other risk factors are excluded [17].
3. Alcoholic AP is characterized by a history of heavy alcohol intake (> 80 mL) within 24 hours before onset or a history of long-term alcohol intake (> 1 year) of > 48 g/day, while other risk factors are excluded [18].
4. Other types of AP refer to acute pancreatitis caused by causes other than biliary, hyperlipidemic, alcoholic, or pancreatitis with complex etiology [19].

The classification of disease severity [1] for AP is as follows:

1. MAP: Patients with neither local complications nor organ failure.
2. MSAP: Patients with brief organ failure or local complications, or both, with a duration of less than 48 hours.
3. SAP: Patients with persistent organ failure, with a duration of more than 48 hours, or those with pancreatitis characterized by one or more local complications.

Extra-pancreatic infections were defined as the presence of infection in at least one site outside the pancreas, confirmed or ruled out through multiple cultures from different sites. Common sites of infection include the respiratory tract, bloodstream, abdominal cavity, biliary tract, urinary tract, and the presence of *Clostridium difficile* in feces [20].

### Statistical analysis

The clinical data of patients with AP were analyzed using SPSS 26.0 statistical software. The study included a description of demographics, disease types, interventions, and types of antibiotics used. Normally distributed measures were expressed as mean ± standard deviation and compared by t-test. Non-normally distributed data were presented using the median and interquartile range (IQR) and analyzed using the Mann-Whitney U test. The comparison of categorical variables was performed using the Chi-square test or Fisher’s exact test. *P* < 0.05 was considered statistically significant.

## Results

### Comparison of baseline demographic, clinical, and laboratory characteristics between the antibiotic and no-antibiotic groups

The study included patients with a mean age of 50.1 ± 16.2 years, of whom 65.8% were male. Among the participants, 681 individuals (60%) received antibiotics during their hospitalization. There were no significant differences in age and gender distribution between the antibiotic-using group and the non-antibiotic-using group. The mean body mass index of the patients was 25.6 ± 4.3, and this parameter did not significantly differ between the two groups. The most prevalent causes of AP were gallstones (57.6%), followed by hyperlipidemia (31.1%), alcohol-related factors (6.2%), and a combination of several causes (4.9%). The distribution of these etiological factors did not significantly vary between the two groups (*P* > 0.05). Upon admission, patients with AP who received antibiotics exhibited higher levels of PCT (4.7 ± 10.8 vs 1.1 ± 2.6, *P* < 0.001), CRP (144.2 ± 125.2 vs 89.4 ± 102.4, *P* < 0.001), serum amylase (954.6 ± 1316.8 vs 693.0 ± 827.3, *P* < 0.003), total bilirubin (30.4 ± 36.8 vs 26.8 ± 24.1, *P* < 0.021), blood creatinine (92.7 ± 79.2 vs 73.4 ± 40.0, *P* < 0.001), and triglycerides (8.3 ± 15.0 vs 5.4 ± 9.3, *P* < 0.001) compared to the non-antibiotic group. Additional relevant baseline characteristics are provided in Table 1.

**Table 1:** Comparison of baseline demographic, clinical, and laboratory characteristics between the antibiotic and no-antibiotic groups

	Total (*n* = 1134)	Antibiotics (*n* = 681)	None (*n* = 453)	*P* value
Age,years	50.1 ± 16.2	50.5 ± 16.4	49.5 ± 16.0	0.247
Male,n(%)	747(65.8)	447(65.6)	300(66.2)	0.445
BMI Index (kg/m^2^)	25.6 ± 4.3	25.6 ± 4.4	25.2 ± 4.1	0.548
Etiology				
Biliary,n(%)	654(57.6)	388(59.3)	266(40.7)	0.560
Hyperlipidemic,n(%)	353(31.1)	211(59.8)	142(40.2)	0.897
Alcoholic,n(%)	71(6.2)	37(52.1)	34(47.9)	0.158
Other,n(%)	56(4.9)	45(52.1)	11(19.6)	0.200
Severity Grade				
Mild,n(%)	459(40.4)	132(19.3)	327(72.1)	< 0.001^*^
Moderate-severe,n(%)	429(37.8)	322(47.2)	107(23.6)	< 0.001^*^
Severe,n(%)	246(21.6)	227(33.3)	19(2.4)	< 0.001^*^
Comorbidity				
HBP,n(%)	383(33.7)	230(60.1)	153(39.9)	0.492
DM,n(%)	224(19.7)	148(66.1)	76(33.9)	0.023^*^
Smoking,n(%)	355(31.2)	229(33.6)	126(66.4)	0.220
Drinking,n(%)	352(31.0)	216(61.4)	136(38.6)	0.295
Laboratory indicators on admission				
Leukocyte count(×10^9^/L)	14.2 ± 5.3	14.7 ± 5.3	13.3 ± 5.1	0.338
Pct(ng/ml)	3.6 ± 9.2	4.7 ± 10.8	1.1 ± 2.6	< 0.001^*^
CRP(μg/ml)	140.0 ± 117.9	144.2 ± 125.2	89.4 ± 102.4	< 0.001^*^
Platelet count(×10^9^/L)	204.0 ± 67.3	206.9 ± 65.3	203.9 ± 68.8	0.560
Serum amylase(U/L)	866.0 ± 1158.0	954.6 ± 1316.8	693.0 ± 827.3	0.003^*^
Alanine aminotransferase(U/L)	71.7 ± 110.0	73.4 ± 114.0	69.3 ± 106.0	0.341
Total bilirubin(μmol/L)	29.0 ± 32.3	30.4 ± 36.8	26.8 ± 24.1	0.021^*^
Creatinine(μmol/L)	85.1 ± 67.0	92.7 ± 79.2	73.4 ± 40.0	< 0.001^*^
Calcium(mmol/L)	2.1 ± 5.2	2.2 ± 6.8	2.0 ± 0.1	0.203
Glucose(mmol/L)	9.3 ± 4.7	9.1 ± 4.8	9.1 ± 4.8	0.945
Potassium(mmol/L)	3.8 ± 0.6	3.9 ± 0.6	3.8 ± 0.5	0.469
Triglycerides(mmol/L)	7.2 ± 13.4	8.3 ± 15.3	5.4 ± 9.3	< 0.001^*^
Modified Marshall score	0(0–8)	0(0–6)	0(0–8)	0.469
BISAP score	1(0–6)	2(0–5)	0(0–4)	< 0.001^*^
APACHE II score	5(0–29)	7(0–29)	5(0–29)	0.348
CTSI score	4(0–8)	4(0–8)	3(0–8)	< 0.001^*^
Laparotomy,n(%)	47(4.1)	36(5.3)	11(2.4)	0.012^*^
Length of hospital stay,(days)	24.8 ± 23.5	28.9 ± 24.5	29.4 ± 26.1	0.753
In-hospital mortality,n(%)	34(2.99)	28(4.1)	6(1.3)	0.735

^*^P < 0.05

### Comparison of baseline demographic, clinical, and laboratory characteristics between antibiotic and no antibiotic groups in AP of different etiologies and disease severity

In patients diagnosed with biliary AP, the average age was 56.1 ± 16.4 years, and 59.9% of the patients were male. Among these patients, 388 individuals (59.32%) were administered antibiotics during their hospital stay. There were no notable differences in age and gender between the group of patients who received antibiotics and those who did not. The mean BMI of the patients was 24.7 ± 4.1, and there was no significant difference observed between the two groups. Regarding admission laboratory indicators, patients with AP who received antibiotics exhibited higher levels of PCT (3.8 ± 7.9 vs. 1.2 ± 3.2, *P* < 0.001), CRP (120.4 ± 103.9 vs. 79.5 ± 81.6, *P* < 0.001), serum amylase (1229.02 ± 1132.42 vs. 902.5 ± 940.4, *P* < 0.039), blood creatinine (93.0 ± 73.2 vs. 74.2 ± 39.1, *P* < 0.001), and blood calcium (2.0 ± 0.2 vs. 2.0 ± 0.1, *P* < 0.001) compared to patients in the non-antibiotic group. The remaining baseline values did not differ significantly between the two groups (Table 2).

**Table 2:** Comparison of baseline demographic,clinical and laboratory characteristics between antibiotic and non-antibiotic groups in acute pancreatitis of different etiologies

		Total	Antibiotics	None	*P* value
Biliary pancreatitis		*n* = 650	*n* = 386	*n* = 264	
	Age,years	56.1 ± 16.4	57.3 ± 16.1	54.2 ± 16.7	0.835
	Male,n(%)	392(59.9)	149(38.0)	113(42.4)	0.296
	BMI Index (kg/m^2^)	24.7 ± 4.1	24.9 ± 4.1	24.4 ± 4.0	0.733
	Smoking,n(%)	153(23.3)	98(25.0)	55(20.6)	0.621
	Drinking,n(%)	139(21.2)	89(22.9)	50(18.7)	0.984
	Comorbidity				
	HBP,n(%)	234(35.7)	142(36.5)	92(34.5)	0.120
	DM,n(%)	83(12.6)	55(14.1)	28(7.2)	0.982
	Severity Grade				
	Mild,n(%)	273(41.7)	69(25.2)	204(74.7)	< 0.001^*^
	Moderate-severe,n(%)	263(40.2)	212(80.6)	51(19.3)	< 0.001^*^
	Severe,n(%)	118(18.0)	11(9.3)	107(90.6)	< 0.001^*^
	Laboratory indicators on admission				
	Leukocyte count(×10^9^/L)	14.3 ± 5.6	15.9 ± 5.7	13.1 ± 4.1	0.325
	Pct(ng/ml)	2.9 ± 6.8	3.8 ± 7.9	1.2 ± 3.2	< 0.001^*^
	CRP(μg/ml)	105.0 ± 98.0	120.4 ± 103.9	79.5 ± 81.6	< 0.001^*^
	Platelet count(× 10^9^/L)	200.8 ± 67.5	120.4 ± 103.9	203.5 ± 64.8	0.840
	Serum amylase(U/L)	1096.1 ± 1069.8	1229.0 ± 1132.4	902.5 ± 940.4	0.039^*^
	Alanine aminotransferase(U/L)	100.0 ± 130.3	107.7 ± 133.4	88.9 ± 125.2	0.142
	Total bilirubin(μmol/L)	33.5 ± 34.5	35.3 ± 38.0	30.9 ± 28.5	0.100
	Creatinine(μmol/L)	85.4 ± 62.3	93.0 ± 73.2	74.2 ± 39.1	< 0.001^*^
	Calcium(mmol/L)	2.0 ± 0.2	2.0 ± 0.2	2.0 ± 0.1	< 0.001^*^
	Glucose(mmol/L)	8.3 ± 4.1	8.5 ± 4.2	7.9 ± 3.8	0.527
	Potassium(mmol/L)	3.8 ± 0.5	3.8 ± 0.5	3.5 ± 3.8	0.735
	Triglycerides(mmol/L)	1.7 ± 2.0	1.7 ± 2.0	1.8 ± 2.0	0.998
	Modified Marshall score	0(0–8)	0(0–6)	0(0–8)	0.957
	BISAP score	1(0–6)	2(0–6)	1(0–4)	0.069
	APACHE II score	5(0–29)	6(0–29)	3(0–27)	0.990
	CTSI score	4(0–8)	4(0–8)	3(0–8)	< 0.001^*^
	Laparotomy,(n%)	30(4.5)	22(5.6)	8(2.0)	0.104
	Length of hospital stay,(days)	23.2 ± 23.7	25.4 ± 24.9	19.9 ± 21.5	0.130
	In-hospital mortality,n(%)	21(3.2)	15(3.8)	6(2.2)	0.105
Hyperlipidemic pancreatitis		*n* = 352	*n* = 211	*n* = 141	
	Age,years	40.6 ± 11.0	39.9 ± 10.8	41.7 ± 11.3	0.418
	Male,n(%)	100(28.3)	67(31.7)	33(23.2)	0.296
	BMI Index (kg/m^2^)	26.5 ± 4.1	27.3 ± 4.8	26.5 ± 4.3	0.204
	Smoking,n(%)	140(39.6)	85(40.2)	55(38.0)	0.087
	Drinking,n(%)	131(37.1)	72(34.1)	59(41.5)	0.675
	Comorbidity				
	HBP, n(%)	106(30.0)	61(28.9)	44(30.9)	0.309
	DM, n(%)	112(31.7)	72(34.1)	40(28.1)	0.767
	Severity Grade				
	Mild, n(%)	139(39.3)	48(22.7)	91(64.0)	< 0.001^*^
	Moderate-severe,n(%)	124(35.1)	78(26.9)	46(32.3)	0.378
	Severe,n(%)	90(25.4)	85(40.2)	5(3.5)	< 0.001^*^
	Laboratory indicators on admission				
	Leukocyte count(×10^9^/L)	14.1 ± 4.7	14.2 ± 4.6	13.9 ± 4.8	0.110
	Pct(ng/ml)	3.9 ± 11.0	5.6 ± 13.4	0.8 ± 1.2	0.086
	CRP(μg/ml)	185.0 ± 123.3	206.1 ± 125.3	152.3 ± 112.9	0.586
	Platelet count(×10^9^/L)	213.5 ± 67.7	215.0 ± 69.4	211.3 ± 65.2	0.355
	Serum amylase(U/L)	521.0 ± 1344.0	621.0 ± 1694.4	377.4 ± 501.9	0.550
	Alanine aminotransferase(U/L)	26.9 ± 23.1	25.3 ± 17.4	29.4 ± 29.6	0.162
	Total bilirubin(μmol/L)	21.8 ± 29.2	22.9 ± 36.3	20.1 ± 12.3	0.391
	Creatinine(μmol/L)	81.7 ± 57.3	88.9 ± 77.9	70.9 ± 45.6	0.125
	Calcium(mmol/L)	2.4 ± 9.4	2.7 ± 12.2	2.1 ± 0.2	0.365
	Glucose(mmol/L)	10.9 ± 5.1	12.1 ± 5.4	9.2 ± 3.9	0.039^*^
	Potassium(mmol/L)	3.9 ± 0.6	3.9 ± 0.7	3.9 ± 0.5	0.119
	Triglycerides(mmol/L)	19.3 ± 97.1	17.0 ± 21.3	10.0 ± 12.8	0.864
	Modified Marshall score	0(0–6)	0(0–6)	0(0–2)	0.043^*^
	BISAP score	1(0–4)	1(0–4)	0(0–3)	0.269
	APACHE II score	6(0–25)	8(0–25)	2(0–17)	0.536
	CTSI score	4(1–8)	5(2–8)	3(1–8)	0.524
	Laparotomy, n(%)	8(2.26)	7(3.3)	1(0.7)	0.999
	Length of hospital stay,(days)	26.2 ± 19.9	33.1 ± 20.9	15.9 ± 12.7	0.130
	In-hospital mortality, n(%)	6(1.6)	6(2.8)	0(0)	0.999
Alcoholic pancreatitis		*n* = 71	*n* = 37	*n* = 34	
	Age,years	46.7 ± 11.8	46.6 ± 10.9	46.8 ± 12.9	0.997
	Male,n(%)	10(14.0)	5(13.5)	5(14.7)	0.999
	BMI Index(kg/m^2^)	26.2 ± 3.7	26.5 ± 4.1	25.8 ± 3.4	0.995
	Smoking,n(%)	39(54.9)	25(67.5)	14(41.1)	1
	Drinking,n(%)	71(100)	37(100)	34(100)	0.999
	Comorbidity				
	HBP,n(%)	12(39.4)	12(32.4)	16(47.0)	0.999
	DM,n(%)	8(21.1)	8(21.6)	7(20.5)	0.995
	Laboratory indicators on admission				
	Leukocyte count(×10^9^/L)	13.7 ± 4.9	14.2 ± 4.7	13.1 ± 5.2	0.993
	Pct(ng/ml)	4.1 ± 10.7	5.4 ± 13.4	2.0 ± 3.4	0.999
	CRP(μg/ml)	130.7 ± 128.7	140.4 ± 129.7	120.0 ± 129.0	0.999
	Platelet count(× 10^9^/L)	206.1 ± 67.7	203.8 ± 62.9	208.6 ± 73.6	0.996
	Serum amylase(U/L)	697.1 ± 781.6	837.1 ± 916.6	548.7 ± 585.0	0.999
	Alanine aminotransferase(U/L)	46.6 ± 86.5	41.5 ± 97.2	52.2 ± 74.2	0.997
	Total bilirubin(μmol/L)	23.1 ± 15.0	21.0 ± 7.9	25.4 ± 20.0	0.995
	Creatinine(μmol/L)	97.1 ± 100.7	113.1 ± 137.2	79.7 ± 20.1	1
	Calcium(mmol/L)	2.0 ± 0.2	1.9 ± 0.3	2.0 ± 0.1	0.994
	Glucose(mmol/L)	9.0 ± 5.6	10.4 ± 6.9	7.6 ± 3.3	0.999
	Potassium(mmol/L)	3.9 ± 0.5	3.8 ± 0.5	3.7 ± 0.4	0.998
	Triglycerides(mmol/L)	3.5 ± 4.9	2.8 ± 3.1	4.3 ± 6.4	0.994
	Modified Marshall score	0(0–6)	0(0–6)	0(0–3)	0.957
	BISAP score	1(0–4)	1(0–4)	1(0–4)	0.069
	APACHE II score	3(0–18)	6(0–18)	2(0–16)	0.990
	CTSI score	4(1–8)	4(1–8)	3(1–6)	0.997
	Laparotomy, n(%)	10(14.0)	2(5.4)	8(0)	1
	Length of hospital stay,(days)	25.7 ± 29.4	34.8 ± 36.7	15.7 ± 13.0	0.993
	In-hospital mortality,n(%)	4(5.6)	4(21.6)	0(0)	1
Other		*n* = 56	*n* = 45	*n* = 11	
	Age,years	44.48 ± 15.36	44.73 ± 14.88	43.45 ± 17.93	0.835
	Male,n(%)	15(26.78)	13(28.8)	2(18.1)	0.296
	BMI Index(kg/m^2^)	26.42 ± 3.91	26.73 ± 3.93	25.13 ± 3.73	0.820
	Smoking,n(%)	22(39.28)	20(62.5)	2(18.1)	0.621
	Drinking,n(%)	25(44.64)	23(71.8)	2(18.1)	0.984
	Comorbidity				
	HBP,n(%)	12(18.46)	11(34.3)	1(9)	0.120
	DM,n(%)	8(14.28)	7(21.8)	1(9)	0.982
	Laboratory indicators on admission				
	Leukocyte count(×10^9^/L)	13.74 ± 5.64	13.72 ± 5.87	13.83 ± 4.84	0.947
	Pct(ng/ml)	5.00 ± 9.01	5.61 ± 9.46	0.64 ± 0.82	0.064
	CRP(μg/ml)	217.12 ± 124.12	234.73 ± 123.09	79.58 ± 81.65	0.186
	Platelet count(×10^9^/L)	194.50 ± 57.24	195.04 ± 58.86	192.27 ± 52.63	0.484
	Serum amylase(U/L)	532.22 ± 505.39	542.38 ± 515.03	486.5 ± 482.58	0.754
	Alanine aminotransferase(U/L)	54.13 ± 121.23	60.07 ± 134.5	29.82 ± 20.57	0.171
	Total bilirubin(μmol/L)	29.04 ± 32.58	31.43 ± 35.84	19.25 ± 7.39	0.117
	Creatinine(μmol/L)	87.84 ± 64.09	91.38 ± 70.41	73.36 ± 22.04	0.097
	Calcium(mmol/L)	1.86 ± 0.38	1.81 ± 0.28	2.06 ± 0.35	0.881
	Glucose(mmol/L)	11.07 ± 4.44	11.68 ± 4.33	8.62 ± 4.23	0.932
	Potassium(mmol/L)	3.97 ± 0.64	3.99 ± 0.67	3.91 ± 0.57	0.938
	Triglycerides(mmol/L)	11.99 ± 11.72	13.47 ± 12.31	6.06 ± 6.47	0.079
	Modified Marshall score	1(0–4)	0(0–6)	0(0–8)	0.196
	BISAP score	2(0–4)	2(0–6)	1(0–4)	0.218
	APACHE II score	8(1–29)	6(0–29)	3(0–27)	0.149
	CTSI score	6(1–8)	4(0–8)	3(0–8)	0.849
	Laparotomy, n(%)	3(4.6)	3(9.3)	0(0)	0.104
	Length of hospital stay,(days)	34.75 ± 30.34	25.46 ± 24.98	19.97 ± 21.51	0.574
	In-hospital mortality,n(%)	3(5.35)	3(9.3)0	0(0)	0.105

^*^P < 0.05

In patients with hyperlipidemic AP, the mean age was 40.6 ± 11.0 years, and 28.3% were male. Among these patients, 211 individuals (59.7%) received antibiotics during hospitalization. There were no significant differences in age and gender between the antibiotic-using group and the non-antibiotic-using group. The mean BMI of the patients was 26.5 ± 4.1, and no significant difference was observed between the two groups. The remaining baseline values also showed no significant differences between the two groups (Table 2).

Among the 71 patients with alcoholic AP, the mean age was 56.1 ± 16.4 years, and 59.9% were male. Out of these patients, 37 individuals with alcoholic AP received early antibiotic treatment, and there were no significant differences in clinical baseline characteristics between the two groups (Table 2).

### Comparison of clinical prognosis between antibiotic and no antibiotic groups in AP of different etiology and disease severity

#### Biliary AP

Table 3 presents the comparative analysis of various outcomes between the group of patients with biliary SAP who received antibiotics and the group without antibiotic treatment. The incidence of laparotomy (*P* < 0.001) and the length of hospital stay were significantly lower (*P* < 0.05) in the antibiotic-treated group. Additionally, a notable difference was observed in the proportion of patients requiring invasive mechanical ventilation, with a lower occurrence in the group receiving antibiotics (*P* < 0.01). However, no significant differences were found in in-hospital mortality, the incidence of AKI, and the need for hemodialysis (Table 3).

**Table 3:** Effect of early antibiotic treatment strategies on prognosis of AP

			Antibiotics	None	*P* value
Biliary Pancreatitis					
	SAP		*n* = 105	*n* = 11	
		Laparotomy, n(%)	22(20.9)	8(72.7)	< 0.001^*^
		Length of hospital stay, days	50.3 ± 33.6	91.0 ± 49.4	0.024^*^
		In-hospital mortality,n(%)	12(11.4)	2(18.2)	0.621
		AKI, n(%)	47(44.7)	6(54.5)	0.546
		CRRT,n(%)	10(9.5)	3(27.3)	0.107
		MV,n(%)	52(49.5)	10(90.9)	0.010^*^
	MSAP		*n* = 212	*n* = 51	
		Length of hospital stay, days	18.2 ± 10.9	91.0 ± 49.4	0.001^*^
		In-hospital mortality,n(%)	0(0)	2(3.9)	0.037^*^
		AKI, n(%)	13(6.1)	3(5.5)	0.947
	MAP		*n* = 69	*n* = 204	
		Length of hospital stay, days	9.07 ± 3.8	12.25 ± 5.0	0.060
Hyperlipidemic pancreatitis					
	SAP		*n* = 85	*n* = 5	
		Laparotomy, n(%)	7(8.2)	1(20)	0.369
		Length of hospital stay, days	44.4 ± 24.0	54.0 ± 39.1	0.075
		In-hospital mortality,n(%)	6(7.1)	0	1
		AKI, n(%)	35(41.2)	0	0.152
		CRRT,n(%)	15(17.6)	0	0.588
		MV,n(%)	40(47.1)	3(60.0)	0.667
	MSAP		*n* = 78	*n* = 46	
		Length of hospital stay, days	30.2 ± 14.7	21.3 ± 9.9	0.064
		In-hospital mortality,n(%)	1(1.2)	0	1
		AKI, n(%)	4(5.1)	3(6.5)	0.710
	MAP		*n* = 48	*n* = 90	
		Length of hospital stay, days	17.9 ± 9.9	16.9 ± 8.3	0.921
Alcoholic Pancreatitis					
	SAP		*n* = 13	*n* = 2	
		Laparotomy, n(%)	2(15.3)	0(0)	1
		Length of hospital stay, days	54.4 ± 56.1	52.5 ± 26.1	0.516
		In-hospital mortality,n(%)	3(23.1)	0(0)	1
		AKI, n(%)	3(23.1)	2(100)	0.095
		CRRT,n(%)	2(15.3)	0(0)	1
		MV,n(%)	10(66.7)	1(50)	0.476
	MSAP		*n* = 15	*n* = 10	
		Length of hospital stay, days	27.4 ± 12.4	21.6 ± 9.1	0.233
		In-hospital mortality,n(%)	1(6.6)	0	1
		AKI, n(%)	1(6.6)	0	1
	MAP		*n* = 9	*n* = 22	
		Length of hospital stay, days	19.0 ± 9.4	9.8 ± 4.1	0.034^*^
Other					
	SAP		*n* = 22	*n* = 1	
		Laparotomy, n(%)	2 (15.3)	0(0)	1
		Length of hospital stay, days	54.4 ± 56.1	–	0.516
		In-hospital mortality,n(%)	3 (23.1)	0(0)	1
		AKI, n(%)	3(23.1)	2(100)	0.095
		CRRT,n(%)	2(15.3)	0(0.0)	1
		MV,n(%)	10(66.7)	1(50)	0.476
	MSAP		*n* = 17	*n* = 0	
		Length of hospital stay,days	27.4 ± 12.4	21.6 ± 9.1	0.233
		In-hospital mortality,n(%)	1(6.6)	0	1
		AKI, n(%)	1(6.6)	0	1
	MAP		*n* = 6	*n* = 10	
		Length of hospital stay,days	15.8 ± 7.8	12.0 ± 5.3	0.317

^*^P < 0.05

Furthermore, among patients with biliary MSAP, the incidence of laparotomy (*P* < 0.001) and the length of hospital stay was significantly lower in the group that received early antibiotics compared to the non-antibiotic group (*P* = 0.037). The proportion of AKI cases did not significantly differ between the two groups (*P* > 0.05) (Table 3).

In the case of patients with biliary MAP, the length of hospital stay was shorter in the group that received early antibiotics; however, the difference was not statistically significant (*P* = 0.06) (Table 3).

#### Hyperlipidemic AP

Table 3 demonstrates that among patients with hyperlipidemic SAP who received early antibiotic treatment, no significant differences were observed in the incidence of laparotomy, in-hospital mortality, length of stay, the incidence of AKI, and the ratio of hemodialysis (*P* > 0.05).

In patients with hyperlipidemic MSAP who received early antibiotic therapy, no significant differences were found in the proportion of patients experiencing in-hospital mortality and the occurrence of AKI about the length of stay (*P* > 0.05).

Furthermore, the length of hospital stay showed no significant difference in patients with hyperlipidemic MAP who were treated with early antibiotics compared to the group not receiving antibiotic treatment (*P* = 0.921) (Table 3).

#### Alcoholic AP

The analysis presented in Table 3 indicates that among patients with alcoholic SAP, no significant differences were observed in the rate of laparotomy, in-hospital mortality, length of stay, the proportion of AKI occurrence, and proportion of invasive mechanical ventilation between the group treated with antibiotics and the group without antibiotic treatment (*P* > 0.05) (Table 3).

Similarly, in patients with alcoholic moderately severe AP (MSAP) who received early antibiotic treatment, no significant differences were found in the length of hospital stay, the proportion of in-hospital mortality, and the occurrence of AKI compared to the group not receiving antibiotics (*P* > 0.05) (Table 3).

Furthermore, the early administration of antibiotics in patients with alcoholic MAP did not significantly improve patient prognosis when compared to the group not treated with antibiotics (Table 3).

#### Other types of AP

The analysis of patients with other types of SAP revealed no significant differences in the rate of laparotomy, in-hospital mortality, length of stay, the proportion of AKI occurrence, and proportion of invasive mechanical ventilation between the group treated with antibiotics and the group without antibiotic treatment (*P* > 0.05) (Table 3).

In patients with other types of MSAP who received early antibiotic treatment, no significant differences were found in the length of hospital stay, the proportion of in-hospital mortality, and the occurrence of AKI when compared to the group not receiving antibiotics (*P* > 0.05) (Table 3).

Furthermore, early administration of antibiotics in patients with other types of MAP did not significantly improve patient prognosis and resulted in prolonged hospital stays (*P* > 0.05) (Table 3).

### Comparison of clinical prognosis of different antibiotic use strategies in AP of different etiology and disease severity

#### Biliary AP

Among the 116 patients diagnosed with biliary SAP, antibiotic therapy was extensively utilized. Specifically, 88 patients were treated with carbapenem antibiotics, while 17 patients received a combination of third-generation cephalosporins and metronidazole anti-infective therapy for biliary SAP. A comparison between the two treatment groups revealed that patients in the carbapenem-treated group experienced a significantly shorter length of hospital stay and a lower in-hospital mortality rate compared to those in the third-generation cephalosporin and metronidazole-treated group at an early stage (*P* = 0.036). Although a trend towards a lower rate of open surgery was observed in the carbapenem-treated group compared to the third-generation cephalosporin and metronidazole-treated group, the difference was not statistically significant (*P* = 0.19). Furthermore, no significant differences were found in the rates of AKI, invasive mechanical ventilation, and hemodialysis between the two treatment groups (*P* > 0.05) (Table 4).

**Table 4:** Effect of different antibiotic use strategies on the prognosis of AP

			Antibiotics			None
			Carbapenem	Third generation cephalosporin	Quinolones	
Biliary Pancreatitis						
	SAP		*n* = 88	*n* = 17		*n* = 11
		Laparotomy,(%)	16(18.2)	5(31.6)		8(72.7)^*▴^
		Length of hospital stay, days	45.8 ± 29.5	71.1 ± 45.5^#^		91.0 ± 49.4^*^
		In-hospital mortality,(%)	9 (10.2)	5 (26.3)^#^		2(18.2)
		AKI,n(%)	39(44.3)	8(42.1)		6(54.5)
		CRRT,n(%)	9(10.2)	1(5.3)		3(27.3)
		MV,n(%)	41(46.6)	11(57.9)		10(90.9)^*^
	MSAP		*n* = 10	*n* = 187	*n* = 15	*n* = 51
		Length of hospital stay, days	32.6 ± 14.6	17.8 ± 10.9	14.2 ± 5.9	36.6 ± 18.4^*▴▪^
		In-hospital mortality,(%)	0	1(0.5)	0	2(3.9)^▴^
		AKI,n(%)	1(10.0)	11(5.9)	1(0.5)	5(9.8)
	MAP		*n* = 2	*n* = 50	*n* = 17	*n* = 204
		Length of hospital stay, days	7.0 ± 1.4	9.3 ± 4.0	8.5 ± 3.2	12.2 ± 5.0^▪^
Hyperlipidemic pancreatitis	SAP		*n* = 20	*n* = 65		*n* = 5
		Laparotomy,(%)	3 (15.0)	4 (6.2)		1(20.0)
		Length of hospital stay, days	47.1 ± 25.2	43.5 ± 23.8		54.0 ± 39.1
		In-hospital mortality,(%)	1(5.0)	5(7.7)		0
		AKI, n(%)	11(55.0)	24(36.9)		0^*^
		CRRT,n(%)	15(17.6)	10(15.4)		0
		MV,n(%)	41(46.6)	11(57.9)		10(90.9)^*^
	MSAP		*n* = 7	*n* = 66	*n* = 5	*n* = 46
		Length of hospital stay, days	41.6 ± 21.0	30.1 ± 15.3	25.8 ± 10.0	21.3 ± 9.9^▴^
		In-hospital mortality,(%)	0	1(1.4)	0	0
		AKI, n(%)	1(14.3)	3(4.5)	0	3(6.5)
	MAP		*n* = 1	*n* = 42	*n* = 5	*n* = 90
		Length of hospital stay, days	–	8.5 ± 3.2	14.0 ± 4.1	11.1 ± 4.4
Alcoholic pancreatitis	SAP		*n* = 9	*n* = 4		*n* = 2
		Laparotomy,(%)	2(22.2)	0		0(0)
		Length of hospital stay, days	65.6 ± 65.1	29.2 ± 10.9		52.5 ± 26.1^▴^
		In-hospital mortality,(%)	3(33.3)	0		0(0)
		AKI, n(%)	4(44.4)	1(25.0)		2(100)
		CRRT,n(%)	2(22.2)	0		0(0)
		MV,n(%)	7(83.3)	2(50.0)		1(50.0)
	MSAP		*n* = 0	*n* = 13	*n* = 2	*n* = 10
		Length of hospital stay, days	–	28.0 ± 12.4	23.0 ± 15.5	0.333
		In-hospital mortality,(%)	–	1(7.6)	1(50.0)	1
		AKI, n(%)	–	1(7.6)	1(50.0)	1
	MAP		*n* = 1	*n* = 7	*n* = 1	*n* = 22
		Length of hospital stay, days	–	18.0 ± 5.7	–	12.2 ± 5.0
Other	SAP		*n* = 10	*n* = 12		*n* = 1
		Laparotomy,(%)	3(33.3)	1(8.3%)		0(0)
		Length of hospital stay, days	47.25 ± 40.49	45.69 ± 27.16		–
		In-hospital mortality,(%)	0(0.0)	1(8.3%)		0(0)
		AKI, n(%)	7(70.0)	6(50.0)		2(100)
		CRRT,n(%)	2(20.2)	0		0(0.0)
		MV,n(%)	8(80.0)	9(75.0)		1(50)
	MSAP		*n* = 0	*n* = 17	*n* = 0	*n* = 0
		Length of hospital stay, days	–	–	–	–
		In-hospital mortality,(%)	–	–	–	–
		AKI, n(%)	–	–	–	–
	MAP		*n* = 3	*n* = 2	*n* = 1	*n* = 10
		Length of hospital stay, days	21.3 ± 6.0	18.0 ± 5.7	–	12.0 ± 5.3

^*^Comparison of the carbapenem group with the no antibiotic group, *P* < 0.05; ^▴^:Triple cephalosporin combined with metronidazole versus no antibiotic group, *P* < 0.05;^#^:Comparison of carbapenems and triple cephalosporins combined with metronidazole group, *P* < 0.05;▪:Quinolone combined with metronidazole versus no antibiotic group, *P* < 0.05

A total of 212 patients diagnosed with biliary MSAP received early antibiotic therapy. Among them, 10 patients were treated with carbapenem antibiotics, 187 patients received a combination of third-generation cephalosporin and metronidazole, and 15 patients received a combination of quinolones and metronidazole. When comparing the subgroups based on antibiotic treatment in biliary MSAP, the carbapenem group exhibited a longer duration of hospitalization compared to the other antibiotic groups (32.6 ± 14.6 vs. 17.5 ± 10.1, *P* = 0.013). Conversely, the group treated with third-generation cephalosporin combined with metronidazole showed a significantly shorter length of hospital stay (*P* = 0.018). No significant reduction in hospital stay was observed with the early use of quinolone combined with metronidazole (*P* = 0.143). Additionally, there were no significant differences in in-hospital mortality and the incidence of AKI between the two groups (Table 4).

A total of 69 patients diagnosed with biliary MAP received early antibiotic therapy. Among them, 50 patients received a combination of third-generation cephalosporin and metronidazole, 17 patients received a combination of quinolones and metronidazole, and two cases were treated with carbapenems. There were no significant differences observed in the reduction of hospital stay between the third-generation cephalosporin combined with the metronidazole treatment group and the carbapenem group (*P* = 0.354). Furthermore, no significant differences were found between the carbapenem group and the quinolone combined with the metronidazole group in terms of reducing the length of hospital stay (*P* = 0.773).

There were no significant differences between the third-generation cephalosporin combined with metronidazole treatment group and the quinolone combined with metronidazole treatment group in terms of reducing the length of hospital stay (*P* = 0.107) (Table 4).

We conduct a logistic regression analysis on the selection of antibiotics most suitable for biliary SAP, early administration of antibiotics can reduce the in-hospital mortality rate (*P* < 0.05) and laparotomy(*P* < 0.05) (Table 5). Through regression analysis on the selection of antibiotics most suitable for biliary SAP, we found that the in-hospital mortality rate in patients using carbapenem antibiotics was half that of patients using third-generation cephalosporins (Table 6).

**Table 5:** Biliary logistic regression analysis of prognosis for antibiotics

	Adjusted OR (95%CI)	*P* adjusted
	Antibiotics	
In-hospital mortality	0.154 (0.033–0.713)	< 0.017^*^
Laparotomy	0.017(0.002–0.167)	< 0.001^*^
MV	0.220 (0.039–1.246)	0.087
AKI	1.528(0.382–2.623E+ 36)	0.549
CRRT	0.971 (0.000–1.246)	0.971

* *p* < 0.05

**Table 6:** Biliary SAP logistic regression analysis of prognosis for antibiotics

	Adjusted OR (95%CI)	*P* adjusted
	Antibiotics	
In-hospital mortality	0.005 (0.000–0.676)	0.03^*^
Laparotomy	0.086 (0.015–0.485)	0.005^*^
MV	0.112 (0.012–1.065)	0.057
	Antibiotics	
In-hospital mortality		
Third generation cephalosporin	1.286(0.194–8.534)	0.795
Carbapenem	0.026(0.001–0.581)	0.021^*^
Laparotomy		
Third generation cephalosporin	0.088(0.010–0.803)	0.031^*^
Carbapenem	0.069(0.010–0.497)	0.008^*^
MV		
Third generation cephalosporin	0.129(0.011–1.154)	0.103
Carbapenem	0.135(0.013–1.457)	0.099

*P* adjusted: assessed by binary logistic regression; adjusted for age, gender, body mass index, hypertension, diabetes mellitus, white blood cell, C-reactive protein, procalcitonin, serum amylase, alanine aminotransferase, platelet, total bilirubin, glucose, serum creatinine. *P < 0.05 was considered statistically significant

#### Hyperlipidemic AP

A total of 85 patients diagnosed with hyperlipidemic SAP received early antibiotic therapy, with 20 patients receiving early carbapenem antibiotics and 65 patients receiving a combination of third-generation cephalosporin and metronidazole. Treatment with carbapenem antibiotics during the early phase did not reduced the occurrence of AKI compared to treatment with third-generation cephalosporin combined with metronidazole. However, there was no significant difference between the two groups in terms of improved mortality. The third-generation cephalosporin combined with metronidazole group had lower rates of open surgery, length of hospital stays, a proportion of mechanical ventilation during treatment, AKI, and hemodialysis compared to the carbapenem group, although statistical significance was not achieved. The use of carbapenem antibiotics in early treatment for hyperlipidemic SAP did not provide additional benefits to the patients (Table 4).

A total of 78 patients diagnosed with hyperlipidemic MSAP received early antibiotic therapy. Among them, seven patients were treated with carbapenem antibiotics, 66 patients received third-generation cephalosporins combined with metronidazole, and five patients received quinolones combined with metronidazole. There were no significant differences observed in the length of hospital stay and the occurrence of AKI between the early use of carbapenem antibiotics and the third-generation cephalosporin combined with the metronidazole group (*P* > 0.05). Furthermore, no significant differences were found in the length of hospital stay and the occurrence of AKI compared to the quinolone combined with the metronidazole group (*P* = 0.316). Additionally, there were no significant differences in the length of hospital stay and the occurrence of AKI between the third-generation cephalosporin combined with metronidazole group and the quinolone combined with metronidazole group (*P* = 0.38) (Table 4).

In the case of hyperlipidemic MAP, a total of 48 patients received early antibiotic therapy. Among them, five patients were treated with quinolones combined with metronidazole, 42 patients received third-generation cephalosporin combined with metronidazole, and one patient received carbapenem antibiotics. There was no significant difference in the length of hospital stay between the group treated with third-generation cephalosporin combined with metronidazole and the group treated with quinolone combined with metronidazole (*P* = 0.461) (Table 4).

#### Alcoholic AP

Thirteen patients diagnosed with alcoholic SAP received early antibiotic therapy during their illness. Among them, nine patients were treated with carbapenem antibiotics, and four patients received a combination of third-generation cephalosporin and metronidazole. The therapeutic effect of early application of carbapenem antibiotics and third-generation cephalosporin combined with metronidazole on improving patient prognosis showed no significant difference between the two groups (*P* > 0.05) (Table 4).

In the case of alcoholic MSAP, a total of 15 patients received early antibiotic therapy. Thirteen patients were treated with third-generation cephalosporin combined with metronidazole, while two patients received quinolone combined with metronidazole. There were no significant differences observed in the length of hospitalization, in-hospital mortality, or the occurrence of AKI between the third-generation cephalosporin combined with metronidazole group and the quinolone combined with metronidazole group (*P* > 0.05) (Table 4).

Nine patients diagnosed with alcoholic MAP received early antibiotic therapy. Among them, one patient received carbapenem antibiotics, seven patients were treated with third-generation cephalosporin combined with metronidazole, and one patient received quinolone combined with metronidazole. Compared to the group not treated with antibiotics, the early use of antibiotics did not significantly improve the prognosis of patients but instead resulted in prolonged hospital stays (Table 4).

#### Other types of AP

Out of the total 22 patients diagnosed with other types of SAP, early antibiotic therapy was administered to all but one patient. Among them, 10 patients received carbapenem antibiotics, while the remaining 12 patients were treated with a combination of third-generation cephalosporin and metronidazole.

For other types of MSAP, a total of 20 patients received early treatment with third-generation cephalosporin combined with metronidazole antibiotics.

Six patients diagnosed with other types of MAP underwent early antibiotic therapy. Among them, three patients were treated with carbapenem antibiotics, and the remaining three patients received a combination of third-generation cephalosporin and metronidazole.

## Discussions

International guidelines [1, 11, 21, 22] generally do not recommend the prophylactic use of antibiotics for the treatment of AP, because AP is not accompanied by infection in most cases, and prophylactic use of antibiotics can increase the risk of bacterial resistance and adverse reactions. However, in some high-risk populations [23], such as those with gallstones, there may be complications of infection, and the use of antibiotics for prophylaxis may need to be considered. Although international guidelines [1, 24] do not recommend prophylactic antibiotics, epidemiology shows that [25] the rate of prophylactic antibiotic use in acute pancreatitis is 60–80%. The rate of prophylactic antibiotic use in our study was higher, which may be due to the 59.6% rate of acute pancreatitis of moderate to severe severity and above. We have added this part to the discussion. Extra-pancreatic infections are associated with severity and local complications in acute pancreatitis, In some instances, antibiotics are administered due to suspicion of EPI [12]. Chinese guidelines recommend the prophylactic use of antibiotics in high-risk populations based on clinical conditions but do not specify the initiation time and course of treatment. This means that clinicians need to make judgments and decisions based on the specific conditions of their patients to avoid unnecessary antibiotic treatment. Although some studies [26–28] have shown that early use of antibiotics in specific disease classifications can alleviate patient symptoms and improve treatment efficacy, early antibiotic treatment remains controversial [29–32]. This is because early use of antibiotics may disrupt the balance of gut microbiota [33].further leading to gut dysbiosis and increased antibiotic resistance [34, 35].

In the early stage of AP, it is difficult to obtain evidence of pathogenic microorganisms in the pancreas and surrounding areas, so the therapeutic role of prophylactic use of antibiotics has always been controversial. guidelines [36] also only support the use of antibiotics in “suspected cases of infected necrotizing pancreatitis” and consider taking further intervention measures. There are also widespread clinical issues of poor compliance with guidelines for the use of antibiotics worldwide. This is because it is difficult in clinical practice to differentiate infectious complications from aseptic inflammatory states. For example, similar fever and tachycardia can occur in clinical symptoms, and both can lead to an increase in white blood cell count and CRP [37]. Procalcitonin(PCT) [38] plays a certain role in predicting infection in patients with acute pancreatitis. It can be used to identify bacterial infection and inflammation in patients with acute pancreatitis, being able to differentiate between bacterial septicaemia and systemic inflammatory response. The PROCAP study [39] used PCT to guide antibiotic use in patients with acute pancreatitis and showed that it could reduce antibiotic use, especially in patients with mild acute pancreatitis, who are more likely to be able to differentiate between sepsis and systemic inflammatory response by PCT. In this investigation,the threshold for a positive procalcitonin test was 1.0 ng/mL. This is less than the international guideline [40] which typically uses a threshold of 0.25 μg/L and 0.5 μg/L for PCT as a means of guiding the withdrawal of antibiotics in patients with both critical and non-critical illnesses. In our study, patients in the antibiotic group had significantly higher PCT levels compared to the non-antibiotic group, and the magnitude of this value may influence clinicians’ judgment on whether to use antibiotics. Our research can guide the early selection of antibiotics for AP based on etiology and severity classification, reducing the mortality rate of severe AP.

The results of this study indicate that early application of antibiotics in patients with biliary SAP can reduce the rate of laparotomy and shorten the length of hospital stay. After biliary AP logistic regression analysis, these two indicators no longer have statistical significance. Early use of carbapenem antibiotics in patients with biliary SAP has an advantage over third-generation cephalosporins in improving patient mortality. A cohort study by Schwarz M et al. showed that patients with severe pancreatitis with a Ranson score ≥ 3 or with CT showing severe necrosis of the pancreas should receive broad-spectrum antibiotics such as imipenem or fluoroquinolones. A single-center study by Nordback et al. [27] found that prophylactic treatment with imipenem and cilastatin reduced mortality (8% vs. 15%), surgical rates (8% vs. 36%), and the total number of major organ complications (28% vs. 76%). Sainio et al. [41] proposed that prophylactic cefuroxime significantly reduced mortality (3.3% vs. 23.3%)in patients with alcoholic necrotizing pancreatitis. However, it is important to note that the total number of cases in this study was small, and the two deaths in the control group occurred 2 and 4 days after diagnosis, respectively, so these deaths are not considered to be due to pancreatic necrosis infection. Several previous studies have argued against the prophylactic use of antibiotics, suggesting that such use does not reduce the likelihood of infected pancreatic necrosis (IPN), mortality, or the need for surgical intervention. However, none of these studies classified and compared the etiology and severity of AP. Therefore, a more detailed classification is needed to determine whether antibiotics are needed at all. In addition, the number of alcoholic SAP cases in our study was small, and a larger sample size is needed to further evaluate the efficacy.

In this study, we found that early antibiotic treatment shortened the hospital stay in patients with biliary MAP and MSAP, but not in patients with non-biliary MAP and MSAP. Biliary tract infections (BTIs) [42–44], including cholangitis and cholecystitis, are common causes of bacteremia. BTIs are associated with a mortality rate of 9–12%. Bacteria are found in the bile of about 75% of patients with acute cholangitis, the most common gram-negative enteric rods; *Escherichia coli* and Klebsiella can account for up to 88%. Prolonged biliary obstruction can lead to sepsis and even death. When selecting antibiotics for the treatment of AP, it is important to consider their ability to penetrate both the bile and gallbladder walls as well as the pancreatic tissue. Previous studies have demonstrated that certain antibiotics, including carbapenems, select third-generation cephalosporins, fluoroquinolones, and anti-anaerobic drugs such as metronidazole, have good permeability and bactericidal activity in pancreatic tissue. Interestingly, biliary obstruction does not interfere with the excretion of imipenem in bile, suggesting that carbapenems may be a good choice for anti-infective therapy in SAP patients with biliary obstruction.

In this study, it was also observed that there was no significant improvement in mortality and laparotomy rate after early antibiotic treatment in patients with non-biliary mild AP and moderately severe AP. This might be related to the pathogenesis of the disease, the early stage of which is characterized by aseptic inflammation. The pathophysiology of HTGAP [45, 46] is associated with the accumulation of free fatty acids (FFAs) and the activation of inflammatory responses. The pathogenesis of alcoholic pancreatitis remains unclear [47, 48]. Existing studies have demonstrated that alcohol is metabolized by pancreatic acinar and stellate cells to produce oxidative and non-oxidative metabolites and metabolic byproducts. In acinar cells, the effects of alcohol and/or its metabolites lead to increased levels of digestive and lysosomal enzymes. This may promote premature activation of digestive enzymes in the acinar cells, leading to pancreatic injury through autodigestion of the glandular tissue.

However, it is important to consider the potential consequences of the long-term use of broad-spectrum antibiotics, which may lead to the development of drug-resistant bacterial infections [37, 49, 50]. Patients with AP are often treated in the intensive care unit (ICU) during the critical phase, and this is the setting where drug-resistant bacteria are most commonly encountered. Drug-resistant bacterial infections are a major cause of mortality in patients with severe AP. Therefore, to use antibiotics rationally, clinicians need to make timely adjustments based on the results of pathogen culture and drug sensitivity testing.

## Conclusions

**Fig. 1:**
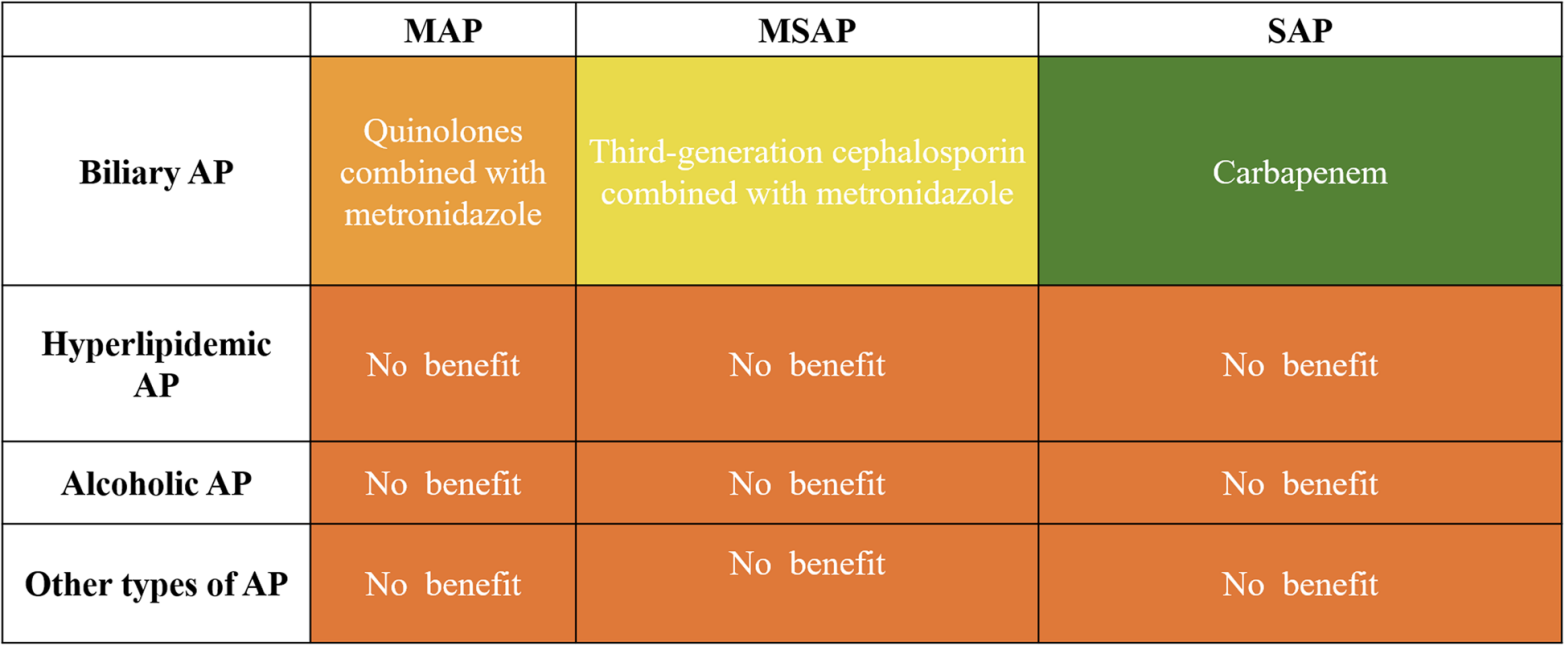
Effect of early antibiotic treatment strategy on the prognosis of AP

Our study showed that early use of carbapenems and third-generation cephalosporin in patients with biliary SAP and MSAP, respectively, improved patient prognosis. Early application of quinolone antibiotics in patients with biliary MAP shortens the length of hospital stay. Early application of antibiotics in patients with non-biliary AP did not significantly improve prognosis. Early antibiotic treatment strategies for AP should be managed according to disease etiology and severity (Fig. 1).

### Limitations

This study has several limitations. It is a single-center retrospective observational study, and the geographical regions represented may not be diverse enough to draw generalizable conclusions. Additionally, the number of cases in some groups was small, which may limit the statistical power of the study. Current research on the use of antibiotics focuses on patients following the development of pancreatic or extra-pancreatic infections, and the use of antibiotics in these cases with clear evidence of infection is less controversial. Our study design focused on the prophylactic use of antibiotics and which antibiotics might benefit patients with acute pancreatitis, stratified according to the etiology and severity of the disease, before clear evidence of infection is available. To the best of our knowledge, this is the first retrospective study in the world to perform a comprehensive stratified analysis. Since the course of treatment in acute pancreatitis is fraught with many variables and the prognosis of the patient is influenced by a multitude of factors, both competent and objective.

Further prospective clinical studies are needed to demonstrate the effect of early antibiotic therapy on the prognosis of AP.

## Data Availability

The data that support the findings of this study are available from the corresponding author upon reasonable request in a de-identified manner.

## Abbreviations

AP: acute pancreatitis
SAP: severe acute pancreatitis
MSAP: moderate severe acute pancreatitis
MAP: mild acute pancreatitis
ERCP: endoscopic retrograde cholangiopancreatography
BMI: Body Mass Index
PCT: procalcitonin
CRP: C-reactive protein
APACHE II: Acute Physiological and Chronic Health Assessment
BISAP: Acute Pancreatitis Severity Index
CTSI: Computed Tomography Severity Index
HTGAP: Hyperlipidemic acute pancreatitis
IQR: interquartile range
BTIs: Biliary tract infections
FFAs: free fatty acids
